# Atrial Fibrillation in Patients with Acute Pulmonary Embolism: Clinical Significance and Impact on Prognosis

**DOI:** 10.1155/2019/7846291

**Published:** 2019-08-19

**Authors:** Katarzyna Ptaszynska-Kopczynska, Izabela Kiluk, Bozena Sobkowicz

**Affiliations:** Department of Cardiology, Medical University of Bialystok, Poland

## Abstract

Pulmonary embolism (PE) is one of the most common causes of cardiovascular death. The most often PE etiology is a deep vein thrombosis (DVT) of the lower extremities, but embolic material can arise in pelvic or upper extremity veins as well as in right heart chambers. There is growing number of evidences of atrial fibrillation (AF) involvement in PE. The presence of AF in patients with PE may be both the cause and the consequence of PE. The PE association with AF should be considered in patients without confirmed DVT and with history of AF, which itself is associated with prothrombotic state. The valuable diagnostic method is echocardiography that may bring the insight into source of embolic material. Another possible AF and PE association is the AF as a consequence of an abrupt increase in pulmonary vascular resistance due to the occlusion of the pulmonary vessels. Large-scale population-based studies have provided a considerable body of evidence on the involvement of PE in the onset of subsequent AF. Another important issue is the influence of AF on prognosis in patients with PE. Most investigators demonstrated a negative impact of AF on mortality. The main problem to resolve is whether AF is an independent mortality risk factor or whether it occurs as a result of comorbidities or the severity of a PE episode. Although the pathophysiological basis of this bidirectional relationship exists, many questions are still unresolved and require further studies, including the significance of paroxysmal AF accompanying an acute PE episode, the usefulness of PE risk scales in patients with concomitant AF, and the effect of anticoagulant treatment on PE and AF occurrence. Regardless of the type of AF, clinicians should be alert to the possibility of PE in patients with previous history of AF or presenting with new-onset AF.

## 1. Introduction

Pulmonary embolism (PE) is one of the most common causes of cardiovascular death. Most often, PE is a complication of deep vein thrombosis (DVT) of the lower extremities, but emboli may also occur in pelvic or upper extremity veins as well as in right heart chambers. Atrial fibrillation (AF) is the most common cardiac arrhythmia affecting a growing number of adults. It is associated with a substantial risk of mortality and morbidity, in large part related to thromboembolic complications [1]. The prevalence of AF in the general population is reported to be 2% to 3% [1, 2]. The important question is whether there in fact is an association between PE and AF or whether they just coexist without any causal relationship. Both conditions are associated with similar risk factors, such as obesity, hypertension, heart failure, or myocardial infarction [1]. Moreover, the risk of PE and AF is strongly related to age.

The involvement of AF in the pathomechanism of PE has not been fully elucidated, and scientific evidence is indirect and based on retrospective observational studies [1, 3, 4]. Also, the impact of AF on prognosis in PE has not been well explained [4]. The presence of AF in patients with PE may be both the cause and the consequence of PE. A bidirectional association between AF and PE was reported previously [5]. Moreover, patients with AF and PE appear to have worse clinical condition as well as higher in-hospital and long-term mortality [6]. Therefore, in this article we would like to summarize the most important findings related to the relationship between AF and PE.

## 2. Atrial Fibrillation as a Risk Factor for Pulmonary Embolism

The association between AF and PE has pathophysiological bases. Atrial fibrillation leads to platelet and coagulation cascade activation along with disordered fibrin turnover, promoting procoagulant state [7, 8]. Blood stasis observed in both atria during AF as well as the anatomical morphology of atrial appendages filled with rough pectinate muscles facilitates thrombus formation. Thrombus formation within the left atrium (LA), particularly in the LA appendage (LAA), and systemic embolization have been well documented in patients with AF [9]. Thus, it may be assumed that thrombus formation in the right atrium (RA), particularly in the RA appendage (RAA), is possible in patients with AF and may cause PE. However, data on such an association are lacking for several reasons. Firstly, the episodes of PE in patients with AF can be silent, whereas systemic embolization in the course of AF is clinically evident and receives increasing attention of scientists and practitioners. Secondly, in patients with PE, most data on the presence of thrombi in the right heart cavities are obtained from transthoracic echocardiography, where the RAA cannot be seen while transesophageal echocardiography is not indicated in patients with confirmed PE [10, 11]. Therefore, it may be assumed that some thrombi localized in the RAA have been overlooked, and the real frequency of RA thrombosis in patients with PE has not been established. Thirdly, most studies concerning right heart thrombosis (RHT) did not report data on the occurrence of AF.

According to the largest population-based autopsy cohort (23796 autopsies), the prevalence of RA thrombosis was the same as of LA thrombosis and reached 3.1% of patients [12]. Moreover, 7% of patients who died from PE had right-sided intracardiac thrombus, and in 62% of them no other potential source of embolization was found. Data according to the presence of AF were not reported. In another autopsy study in patients with AF, LA and RA thrombi were found in 12.6% and 7.5% of cases, respectively [13]. Right heart thrombosis was present only in 2% of patients who died from infectious diseases; however, it was associated with a 6.5-fold higher prevalence of PE [12]. The conclusion from the population-based study is that thrombi in the RA, despite being difficult to assess in clinical practice, occur with a similar frequency as those in the LA [12].

Contrary to the above results, echocardiographic studies showed that thrombi in the RA and RAA have been detected at a frequency of 0.4% to 7.5%, which is much lower than that of thrombi in the LA and LAA detected at a frequency of 10% and 15% [14, 15]. Thrombi in the RA and RAA are frequently accompanied by thrombi in the LA or LAA. Isolated RAA thrombus is very rare and usually occurs in patients with AF accompanied by congestive heart failure or mitral or tricuspid valve stenosis [14, 16, 17]. Cresti et al.[17] evaluated 1042 patients with persistent AF or atrial flutter referred for transesophageal echocardiography-guided cardioversion. The incidence of RAA thrombus was significantly lower (0.75% compared with 10.3% in the LAA). Moreover, RAA thrombi were large and ranged from 12 to 35 mm. In patients with RAA thrombi, the clinical signs of PE were not observed but the possibility of silent PE was not investigated. There are data on the relationship between spontaneous echo contrast within the RA and perfusion defects on pulmonary scintigraphy in patients with nonvalvular AF, suggesting silent PE [18]. However, data concerning the association between the presence of RAA thrombi in AF patients and subsequent PE are lacking.

The discrepancies in the occurrence of RAA thrombi between autopsy studies conducted in the years from 1970 to 1982 and contemporary echocardiographic data may be explained by several reasons, such as different patient populations included in the studies and the time when the investigations were carried out [14]. The common use of long-term anticoagulant treatment in patients with AF in recent decades has probably reduced the risk of thrombosis. The lower frequency of thrombus formation in the RAA compared with the LAA may also result from the differences in morphology and anatomical localization of both appendages. The RAA is unilobar and triangle shaped, with the inner surface composed of a dense network of pectinate muscles (prone to thrombosis) [19]. A broad RAA ostium may facilitate thrombus migration. On the other hand, the location of the RAA adjacent to the inflow of the superior vena cava may reduce the ability of clot formation.

## 3. Epidemiological Data

The prevalence of AF in patients with PE ranges from 15% to 21% and is much higher than that in the general population (2%-3%) [20–22]. Recently, Lutsey et al. [5] investigated a relationship between AF and venous thromboembolism (VTE) in 15129 patients followed for almost 20 years. The occurrence of AF was associated with a significantly higher risk of a subsequent VTE episode (hazard ratio [HR], 1.71 [95% CI, 1.32–2.22]), particularly during the first 6 months after an AF event, as compared with patients in sinus rhythm [5].

The triggering role of AF in the development of PE comes from the observation that patients with PE without concomitant DVT more frequently had a history of AF in comparison with patients with PE related to DVT [23–25]. Keller et al. [23] showed that in patients with isolated PE, persistent and permanent AF were detected markedly more often than in patients with PE and a history of DVT (52.9% and 65.5% versus 47.1% and 34.5%, respectively) [23]. This association was the strongest for permanent AF and the weakest for paroxysmal AF. Therefore, the theory of clot formation in the RA in patients with AF and subsequent PE seems plausible. The association between PE, AF, and RHT and its prognosis has been reported [13, 26]. In a study by Kukla et al. [26], RHT was found in 5% of the whole study group, in 8% of patients with paroxysmal AF, and in 7% of patients with nonparoxysmal AF [26]. In a study by Krajewska et al. [3], the presence of RHT was confirmed in 5.9% of patients in sinus rhythm, with a tendency to a higher prevalence in the groups with paroxysmal AF and permanent AF (10% and 14.3%, respectively). The differences did not reach significance, mainly because of a small population size in both studies. Moreover, it is important that in both studies RHT detection was based on transthoracic echocardiography, so some thrombi may have been overlooked.

## 4. Atrial Fibrillation as a Consequence of Pulmonary Embolism

Data concerning the influence of PE on AF occurrence are ambiguous. There are several possible pathophysiological mechanisms leading to an AF episode in patients with PE. Atrial fibrillation may reflect right ventricular (RV) failure and pressure overload related to an abrupt increase in pulmonary vascular resistance due to the occlusion of the pulmonary vessels [27]. Stretch injuries of RA tissue may facilitate the onset of AF. In patients with PE, a higher prevalence of AF was found when RV function was impaired in comparison with patients with normal RV function (39% versus 12%) [28]. Furthermore, RV enlargement results in tricuspid valve regurgitation, which in turn predisposes to RA dilation and volume overload. Both these pathomechanisms promote the foci for reentry and further AF development [29].

### 4.1. Epidemiological Data

Large-scale population-based studies have provided a considerable body of evidence on the involvement of PE in the onset of subsequent AF [3, 9, 30]. The increased risk of late-onset AF has been observed in patients after acute PE during long-term follow-up [24].

Independent predictors of subsequent AF were age, history of congestive heart failure, obstructive sleep apnea, and low sodium levels at baseline [24]. The relationship between VTE and risk of AF has been investigated in the prospective Tromsø Study during 16 years of follow-up [30]. Investigators found that incident VTE was associated with future risk of AF (HR, 1.63; 95% CI, 1.22–2.17) compared with individuals without VTE. This observation suggests that PE may lead to cardiac dysfunction that may trigger AF. Moreover, the risk of AF was especially high in the first 6 months after the PE event [5]. Contradictory data were published by Martin et al. [31]. They investigated patients with implanted devices, such as implantable cardioverter defibrillator or cardiac resynchronization therapy, who were monitored for atrial tachyarrhythmia. The authors showed no temporal correlation between atrial tachyarrhythmia and clinical thromboembolic events [31].

## 5. Paroxysmal and New-Onset Atrial Fibrillation following an Episode of Pulmonary Embolism

Most studies did not investigate the relationship between PE and different types of AF (paroxysmal, persistent, and permanent). There have been only a few studies published on the occurrence of PE and paroxysmal AF [2, 21, 23, 24]. However, because of the differences in methodology, the results are difficult to compare. There has been only one study that assessed the presence of paroxysmal AF in patients with PE at index hospitalization [3]. Compared with patients with permanent AF and sinus rhythm, those with paroxysmal AF were older and had worse echocardiographic parameters reflecting RV afterload, such as the highest estimated systolic pulmonary artery pressure and the shortest pulmonary artery acceleration time. This may indicate a causal relationship between the severity of PE and an episode of paroxysmal AF [3]. Paroxysmal AF was identified in 8% of patients [3]. Similar frequency (6%) was found in the study by Kukla et al. [26] despite the inclusion into a study group also patients with a previous history of AF. In another study, which included 295 patients with PE from the thrombEVAL study, paroxysmal AF was reported in 13% of participants [23]. Thus, the association between PE and subsequent AF seems to be confirmed, but there are not enough data to prove that the relationship between the severity of a PE episode and paroxysmal AF.

## 6. Influence of Atrial Fibrillation on Prognosis in Patients with Pulmonary Embolism

The exact effect of AF on the outcome in patients with acute PE is not fully understood. Some investigators demonstrated a negative impact of AF on mortality [1, 22, 24]. Barra et al. [22] suggested that patients with a history of AF had higher in-hospital, 1-month, and 6-month mortality rates. Furthermore, the presence of AF at admission showed additive prognostic impact on 1-month and 6-month mortality risk, despite similar in-hospital death rates [22].

Ng et al. [24] also reported a worse prognosis in patients admitted due to PE with baseline or subsequent AF. Patients with AF diagnosed prior to a PE episode had the highest mortality rate in long-term follow-up, with the rates of 23.7% at 1 year and 45.4% at 5 years, compared with patients with no AF (16.7% and 29.5%, respectively) and with those with AF after hospitalization (9.5% and 34.9%, respectively) [24]. On the other hand, contradictory results on the lack of the relationship between AF and prognosis in PE patients have also been reported [20, 23, 32].

The main issue to resolve is whether AF is an independent mortality risk factor or whether it occurs as a result of comorbidities or the severity of a PE episode. Moreover, in the majority of studies, the effect of AF on survival was analyzed without differentiation of AF patterns.

The standard scales used in risk assessment in patients with PE include the heart rate but not the heart rhythm. In a study that applied the Simplified Pulmonary Embolism Severity Index and Geneva scales for risk assessment, both patients with paroxysmal AF and those with permanent AF were significantly more often classified into the high-probability group compared with patients in sinus rhythm [3]. The highest proportion of patients with paroxysmal AF in the high-probability group resulted from the prevalence of tachycardia (>100 beats/min) on admission. Despite the results of both scales, the in-hospital mortality rate of patients with paroxysmal AF was rather low (and similar to that in individuals in sinus rhythm), in contrast to patients with permanent AF in whom the rate was high (6.5% versus 5% versus 25%) [3]. It is possible that the presence of tachycardia associated with paroxysmal AF may be a confounding variable.

## 7. Gaps in Evidence

The main issue that has been less well studied and is thus unresolved is whether the widespread use of anticoagulants to prevent stoke and systemic embolization in AF patients may additionally prevent VTE. Such an observation was partially confirmed in a study where, in patients with AF on oral anticoagulants, subsequent risk of VTE was the lowest for those treated with apixaban (HR (95% CI) 0.51 (0.39–0.68) and dabigatran (0.55 (0.47–0.66)), as compared to warfarin. There was no difference in VTE risk when comparing rivaroxaban to warfarin (1.01. (0.87-1.19)) [33]. Also, in a large cohort study, the risk of AF after a PE episode was attenuated after adjustment for anticoagulant therapy [5]. Recent results from the GARFIELD-AF Registry showed that the incidence of PE was much lower than previously reported: only 2.6% of AF patients had a history of PE or DVT. During follow-up, PE was reported in 4.9% of patients who died within 1 month after the diagnosis of AF, 3.2% in those who died within 2 to 12 months, and only in 2.6% of those who survived 12 months [34]. There are no data on whether anticoagulant therapy used to prevent VTE recurrence may additionally protect against AF.

## 8. Summary

The causal relationship between PE and AF seems to be proved and work in both directions. Patients with AF are at risk of subsequent PE development and vice versa—those with PE are at risk of AF during follow-up. Although the pathophysiological basis of this bidirectional relationship exists, many questions are still unresolved and require further studies, including the significance of paroxysmal AF accompanying an acute PE episode, the usefulness of PE risk scales in patients with concomitant AF, and the effect of anticoagulant treatment on PE and AF occurrence. Regardless of the type of AF, clinicians should be alert to the possibility of PE, particularly in patients presenting with new-onset AF.
